# Exploring Pain Reduction through Physical Activity: A Case Study of Seven Fibromyalgia Patients

**DOI:** 10.3390/bioengineering11080765

**Published:** 2024-07-29

**Authors:** Marit Dagny Kristine Jenssen, Elisa Salvi, Egil Andreas Fors, Ole Andreas Nilsen, Phuong Dinh Ngo, Miguel Tejedor, Johan Gustav Bellika, Fred Godtliebsen

**Affiliations:** 1 Department of Mathematics and Statistics, University of Tromsø—The Arctic University of Norway, NO-9019 Tromsø, Norway; miguel.tejedor@ehealthresearch.no (M.T.); fred.godtliebsen@uit.no (F.G.); 2 Norwegian Centre for E-Health Research, P.O. Box 35, NO-9038 Tromsø, Norway; elisa.salvi@ehealthresearch.no (E.S.); phuong.dinh.ngo@ehealthresearch.no (P.D.N.); johan.gustav.bellika@ehealthresearch.no (J.G.B.); 3 Department of Public Health and Nursing, Norwegian University of Science and Technology (NTNU), NO-7491 Trondheim, Norway; egil.a.fors@ntnu.no; 4 Department of Health and Care Sciences, University of Tromsø—The Arctic University of Norway, NO-9019 Tromsø, Norway; ole-andreas.nilsen@uit.no; 5 Department of Physics and Technology, University of Tromsø—The Arctic University of Norway, NO-9019 Tromsø, Norway; 6 Department of Clinical Medicine, University of Tromsø—The Arctic University of Norway, NO-9019 Tromsø, Norway

**Keywords:** fibromyalgia, physical activity, Fitbit, activity tracker, patient-reported data, data analysis, patient empowerment

## Abstract

Fibromyalgia is a chronic disease that affects a considerable fraction of the global population, primarily women. Physical activity is often recommended as a tool to manage the symptoms. In this study, we tried to replicate a positive result of pain reduction through physical activity. After collecting pain and physical activity data from seven women with fibromyalgia, one patient experienced a considerable reduction in pain intensity. According to the patient, the improvement was related to physical activity. Our study was conducted to investigate the replicability of this result through personalized activity recommendations. Out of the other six patients, three experienced a reduction in pain. The remaining three patients did not experience any pain relief. Our results show that two of these were not able to follow the activity recommendations. These results indicate that physical activity may have a positive effect on chronic pain patients. To estimate how effective physical activity can be for this patient group, an intervention with longer follow-ups and larger sample sizes needs to be performed in the future.

## 1. Introduction

Fibromyalgia (FM) is a chronic disease characterized by widespread pain, fatigue, and poor sleep [1,2,3,4,5]. FM affects 1.7–4% of the population [3,6,7,8], with a higher prevalence in women [9,10,11]. Research has shown that FM has a negative impact on the quality of life of patients [12,13]. Despite extensive research, the causes of FM remain poorly understood, making effective treatment a continuous challenge for both patients and healthcare providers. Improving health for FM patients is important both for the individual and the society. Sick leave and disability benefits for people with FM are huge costs for society [14,15,16,17]. In a systematic review from 2022 [15], it was estimated that the total annual cost per FM patient ranged between USD 1250 and USD 8504 in Europe. Helping FM patients manage their pain and other symptoms might improve their quality of life and reduce their need for sick leave and disability benefits.

Examples of existing pain management options are painkillers, physical activity, massage therapy, and acupuncture [18]. Dietary interventions have also been tested [19,20,21]. In [22], clinical trials on non-pharmacological therapies for adults with FM were reviewed. After reviewing 46 studies, the authors reported strong evidence that non-pharmacological therapies, including physical activity, can be efficient pain management methods. Physical activity is often recommended as a non-pharmacological intervention for pain reduction in people with FM [23,24,25]. A PubMed search for “fibromyalgia AND activity” yielded approximately 3500 results as of May 2024, which indicates that there has been extensive research on physical activity as a tool for managing FM symptoms. A systematic review from 2023 [26] studied five papers on exercise as a treatment option for FM patients [27,28,29,30,31]. All the reviewed papers concluded that physical activity is beneficial for FM patients. Although physical activity is beneficial, patients may struggle to be active. Systematic reviews [32,33] have concluded that people with FM or other rheumatic diseases are less physically active than the general population. In addition, people with FM tend to have a higher fear of movement than apparently healthy subjects [34,35]. A qualitative study [36] interviewed sixteen individuals with chronic pain. They were asked about their experiences, barriers, and facilitators to be physically active. Examples of barriers included fatigue, fear of increased pain and injury, lack of social support and motivation, and mental health challenges.

Physical activity data can be collected using self-reporting, or the study participants may use wearable trackers that automatically collect data on their activity habits. Fitbit produces wearable trackers that can be placed on the user’s wrist [37] and might be a suitable tool to help improve lifestyle in terms of physical activity [38]. Using activity trackers to monitor subjects with FM is not a very common strategy. A PubMed search for “fibromyalgia AND activity tracker” yields only one result [39] where 107 women with FM were included. In that study, moderation analyses showed that the association between physical activity and pain intensity was moderated by pain catastrophizing. A randomized controlled trial [40], with 16 women in the test and control groups, respectively, found that low-intensity physical activity might help reduce pain catastrophizing. Several studies conclude that physical activity recommendations should be individually adapted [41,42,43,44,45].

Based on our literature review, we hypothesized that personalized physical activity recommendations could significantly reduce pain levels in FM patients. Specifically, we aimed to (1) assess changes in pain levels pre- and post-activity, (2) explore the relationship between pain intensity and different variables measured by Fitbit trackers (such as physical activity), and (3) analyze patient responses to a post-program questionnaire in order to assess their experience of being part of our study. By assessing the feasibility of personalized activity recommendations on a small patient group, our study seeks to pave the way for larger studies that could refine our approach to pain management in FM through lifestyle adjustments.

## 2. Materials and Methods

### 2.1. Patient Sample

The participants in this study were recruited through the Norwegian FM Patients Association [46]. Our recruitment efforts resulted exclusively in female respondents, which likely reflects the higher prevalence of FM among women. The study initially involved twelve participants, all Norwegian women, aged between 40 and 66 years.

The recruitment occurred in two phases: participants 1–5 were enrolled at the start of the study. Participant 3 dropped out shortly thereafter. Approximately six months later, a second group of seven participants (participants 6–12) joined the project. However, due to personal reasons, participants 7, 8, 10, and 11 withdrew from the study. Finally, seven participants fully completed the study.

The participants gave informed consent to data collection. They could withdraw their consent at any time, without giving any reason. We followed the recommendations from [47] regarding empowering and informing the participants.

### 2.2. Data Collection

#### 2.2.1. Physical Activity Data

To monitor physical activity levels, participants used Fitbit activity trackers. The specific models employed were as follows:

- Five participants used the Fitbit Charge 4: This model provides advanced health metrics, including heart rate monitoring, step counting, and sleep tracking. It also features GPS tracking for precise measurement of outdoor activities.
- One participant used the Fitbit Charge 3: Similar to the Charge 4 but lacks the built-in GPS feature.
- One participant used the Fitbit Versa 3: This model offers functionalities similar to the Charge 4 but includes additional features such as voice control and the ability to store and play music directly from the watch.

We provided all patients with Fitbit Charge 3 or 4, but one participant decided to wear her own tracker (Versa 3). All models are capable of recording the following data:

- Heart Rate: Continuous monitoring throughout the day and calculating the resting heart rate.
- Steps Counted: Total number of steps taken each day.
- Activity Levels: Categorized as sedentary, lightly active, moderately active, and very active.

Each device provided daily activity summaries, which included the total number of steps per day, distance moved, and minutes spent at various activity intensities. These summaries were crucial for assessing the physical activity patterns of participants and associating them with changes in pain intensity.

#### 2.2.2. Pain Data

Pain intensity was recorded using a mobile application specifically developed for this study. Participants were instructed to register their pain levels three times a day: at 9 a.m., 2 p.m., and 9 p.m. The pain was quantified using the visual analog scale (VAS), a validated continuous measurement instrument that ranges from 0 (no pain) to 100 (worst imaginable pain) [48,49]. This scale is widely used in clinical research for its sensitivity and simplicity, allowing for precise monitoring of pain variations over time.

#### 2.2.3. Data Handling

All data from Fitbit devices and the mobile application were synchronized and stored securely in compliance with data protection regulations. Data anonymization was applied to ensure participants’ privacy before analysis. Additionally, the project had a contact person that the participants could reach out to in case of any technical problems with the activity tracker or the pain registration app.

### 2.3. Statistical Analysis

#### 2.3.1. Correlation

Correlation is a measure of the linear relationship between two variables. The correlation ranges from −1 to +1. If the correlation is equal to or close to zero, there is no linear relationship between the variables. A positive (negative) correlation means that an increase in one variable corresponds to an increase (decrease) in the other one. It is important to note that correlation does not necessarily mean causality.

We calculated the correlation between pain intensity and different Fitbit variables. Since we cannot assume that our data are normally distributed, we apply Spearman’s rank correlation coefficient [50]:

(1) $$\begin{array}{c} r_{s}=1-\frac{6}{n(n^{2}-1)}\sum_{i=1}^{n}d_{i}^{2}, \end{array}$$

where $d_{i}$ is the difference between the ranks assigned to $x_{i}$ and $y_{i}$ and *n* is the number of pairs of data. When we test correlations, we formulate two hypotheses, a null hypothesis and an alternative hypothesis. The null hypothesis is that there is no correlation (correlation equals zero), and the alternative hypothesis is that the correlation is positive (“greater”), negative (“less”), or non-zero (“two-sided”). Before performing a hypothesis test, it is necessary to determine a significance level. The p-value of a test is the probability of obtaining a result at least as extreme given that the null hypothesis is correct. If the p-value is below the predetermined significance level, the null hypothesis is rejected. It is common to set the significance level to 5%, and we also use this significance level in our hypothesis testing. To be specific, we set up the following null hypothesis:

**H_0_.**  *The correlation between pain intensity and any Fitbit variable equals zero.*

The alternative hypothesis for time spent sedentary and resting heart rate is as follows:

**H_*a*_.**  *The correlation between pain and sedentary time/resting heart rate is positive.*

For time spent in the different activity zones (lightly, moderately, and very active), we formulate the following alternative hypothesis:

**H_*a*_.**  *The correlation between pain and time spent in different activity zones is negative.*

Several studies indicate that physical activity is beneficial for FM patients, therefore we perform a one-sided test to check whether we can claim that increased time spent physically active is associated with less pain. Conversely, we want to investigate whether more time spent sedentary and an increased resting heart rate is associated with increased pain intensity. A high resting heart rate is associated with a reduced physical shape, and we believe that there might be an indirect association between resting heart rate and pain intensity.

#### 2.3.2. SiZer

Significant zero crossings of derivatives (SiZer) is a powerful tool for exploring complex patterns in curves and time series [51]. SiZer estimates several functions to a dataset based on different bandwidths. For each bandwidth and in each position, a 95% confidence interval of the derivative is calculated. If zero is included in the confidence interval, the derivative is not significantly nonzero. Otherwise, the derivative is significantly positive or negative. The underlying idea of SiZer is to use many scales because observed relationships can be observed at many different levels of resolution.

The SiZer algorithm that we use in this study assumes that all data points are independent [51,52]. Since this assumption may be violated in some cases, we mainly use SiZer as an exploratory tool for detecting potential relationships. To be specific, we use SiZer as an exploratory tool to check if, for example, increasing minutes of light activity might have any relation to perceived pain. The results should be interpreted with caution due to the independence assumption but will indicate what relationships that could be significant in practice.

Our study did not perform cross-participant statistical analyses due to the small sample size. Instead, we ran individual analyses for each participant to monitor and evaluate the effect of personalized activity interventions on their pain profiles over time. By analyzing each participant’s data separately, we were able to focus on personal outcomes without aggregating data across the group.

### 2.4. Intervention

When we analyzed the data from the first five months of data collection, we observed that one participant (Participant 4) had experienced a considerable reduction in pain. Through a meeting with a physician, she explained that she had started to follow a regular physical activity program. We attempted to replicate this result for the other six participants by inviting them to start a physical activity program (which we will refer to as “intervention”) supervised by a physiotherapist. Two research questions were addressed in this study:

1. Does spontaneous physical activity affect the pain pattern in our research participants?
2. Can the results of one patient’s successful pain reduction intervention be replicated for other FM patients?

**Figure 1:**
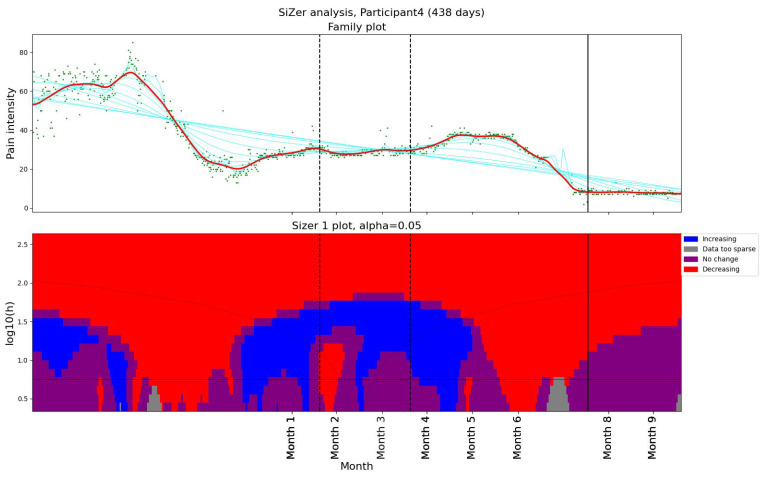
The SiZer plot of the entire pain time series for Participant 4, who experienced pain relief prior to our intervention. The vertical dashed lines indicate the start and end of the baseline period, whereas the vertical solid line is placed at the dates of the meeting with the physiotherapist. Explanation of the panels and colors is given in Section 2.4.

We used SiZer (Section 2.3.2) to assess whether the pain had changed significantly in Participant 4 (Figure 1). The upper panel in Figure 1 shows the data points (green dots) together with a family of “smoothings” as functions of the bandwidth *h* (cyan curves). The thick red curve corresponds to the Sheather–Jones plug-in bandwidth [53]. In the lower panel, a feature map is shown as a function of scale and time. If the derivative is significantly positive (negative), the map is colored in blue (red). If the derivative is not significantly nonzero, the map is colored in purple. When the data are too sparse to draw a conclusion about the derivative, the map is colored gray. The horizontal black line in the lower panel corresponds to the Sheather–Jones plug-in bandwidth.

During two months, the pain level decreased from above 60 on the VAS scale to below 20 (Figure 1). Although no significant change in activity pattern was observed in the Fitbit data, Participant 4 explicitly reported having started regular exercise and treatment, which likely contributed to the reduction in pain. To investigate further, we organized an in-depth interview with the participant, run by a professor specialized in general medicine and psychiatry. In the interview, the patient reported using several methods to reduce pain intensity. Another observation was that Participant 4 was more physically active than the other patients in the study.

We asked the other patients if they would like to start a regular physical activity program (“intervention”). To monitor the effect of the intervention, we defined a “baseline period” and an “intervention period”. In the baseline period, the participants were supposed to wear Fitbit and register pain, but they were not asked to change their lifestyle in any way. We defined the start of the intervention as the date of the first meeting with the physiotherapist regarding improved lifestyle. By collecting data during a baseline period and an intervention period, each participant acted as their own control, with changes observed over time within the same individual. This approach naturally accounts for factors like age and body weight, which remain relatively constant for each participant over the study period. Our baseline and intervention periods are defined as follows:

- Baseline period: the 20th of month 1–20th of month 3.
- Intervention period: the date of the first meeting with the physiotherapist—month 9.

The intervention period started four months after the baseline period ended. The reason for the gap was summer vacation and the lack of availability of the participants. The meetings with the physiotherapist had the following structure:

1. Background: The participants were invited to share their everyday life experiences and challenges related to chronic pain.
2. Data review: Using graphs visualizing activity and pain, the physiotherapist helped the participant understand the relationship between activity and pain.
3. Hypothesis formulation: The participant was encouraged to articulate her hypotheses for why activity might have an impact on chronic pain.
4. Activity evaluation: The physiotherapist evaluated the activities that the participants were already doing spontaneously and challenged them to consider how to improve (e.g., increase/decrease the intensity, increase/decrease the frequency).
5. Patience and care: The physiotherapist emphasized the importance of starting carefully and being patient, as changes take time.
6. Goal setting: The participant and physiotherapist agreed on trying to make a change before the follow-up meeting.

Below is a summary of the goals set during the meetings.

- Participant 1 agreed to try increasing their activity level, dancing, and using stairs.
- Participant 2 liked walking in the woods. The plan was to increase activity level, but starting slowly.
- Participant 4 was already active and used to the activity. The plan was to continue her current activities.
- Participant 5 planned to resume walking, making sure to increase heart rate.
- Participant 6 was already used to being very active with various exercise equipment.
- Participant 9 was active with hiking and swimming. The plan was to continue her current activities.
- Participant 12 had a morning stretch routine and hiked regularly. The plan was to increase hiking speed to increase heart rate.

As described above, the design of our study involved personalized activity recommendations rather than a homogeneous activity program. This decision was based on the findings from previous studies (referred to in the introduction) that exercise programs for FM patients should be individually adapted.

### 2.5. Questionnaire

At the end of the intervention, we sent out a questionnaire asking the patients about their experience of being part of this study. The questionnaire consisted of 11 questions (listed below), mostly answered using a 5-point Likert scale [54]: 1 corresponds to strongly disagree/very unsatisfied and 5 corresponds to strongly agree/very satisfied. For questions 1, 4, and 5—1 corresponds to “uncomfortable and would not like to continue/do it again”, whereas 5 corresponds to “comfortable and would like to continue/do it again”. Questions 9 and 10 are answered by 1, which corresponds to “yes” or 0, corresponding to “no”. The participants are also invited to elaborate on their responses. The entire questionnaire with all choices is included as an appendix.

1. How did you feel about wearing a Fitbit and registering pain intensity 3 times per day for the duration of this project?
2. How would you rate your level of satisfaction with the Fitbit tracker?
3. How would you rate your level of satisfaction with the pain registration app?
4. How did you feel about the fact that the meeting with the physiotherapist was digital, instead of in person?
5. How did you feel about the physical activity plan that you have created with the physiotherapist?
6. Was it useful to discuss the graphs that were provided during the meeting?
7. Do you agree with this affirmation: “I think that the personalized activity plan that I followed has helped me improve my pain management”?
8. If you do not agree with the affirmation above: What do you think are the reasons the personalized activity plan did not work for you?
9. If offered, would you like to continue with the “assisted” personalized activity intervention?
10. We are considering keeping the app up and running for 6 extra months. It would be possible to register pain 3 times a day and collect Fitbit data. However, consultations with the physiotherapist would not be available. Would you be willing to collect data for us?
11. Help us improve the project. Do you have any input to share with us?

## 3. Results

### 3.1. Observed Relationships

We tested the correlation between maximum pain and activity summaries during one day. In addition, we looked at the correlation between maximum pain and resting heart rate. Significant results are shown in Table 1. Four participants (1, 2, 9, and 12) have a significant positive correlation between maximum pain and resting heart rate. Participant 2 exhibits a significant negative correlation between maximum pain and both minutes lightly active and the number of steps. Participant 9 has a significant positive correlation between pain and minutes spent sedentary, and Participant 12 has a significant negative correlation between pain and minutes spent very active. The other correlations were not significant. This does not necessarily mean that there are no additional relationships between pain and other Fitbit data.

### 3.2. Pain and Activity Patterns

#### 3.2.1. Changes in Pain Levels

**Figure 2:**
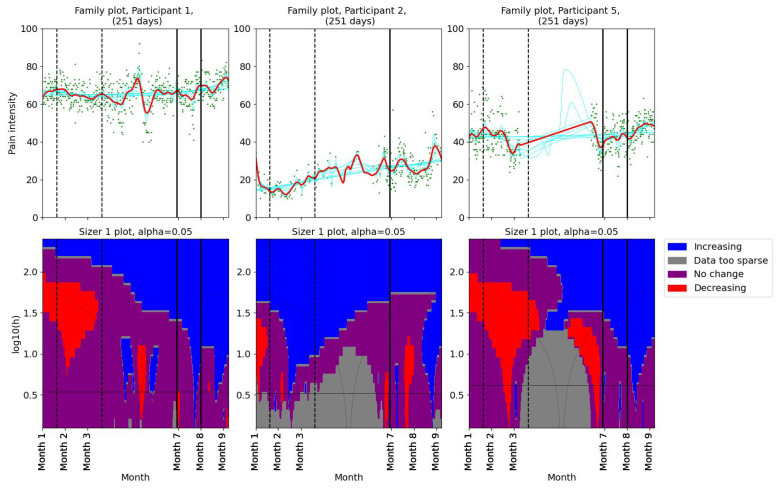
SiZer plots of the pain time series for participants 1, 2, and 5. The vertical dashed lines indicate the start and end of the baseline period, whereas the vertical solid lines are placed at the dates of the meetings with the physiotherapist. Explanation of the panels and colors is given in Section 2.4.

**Figure 3:**
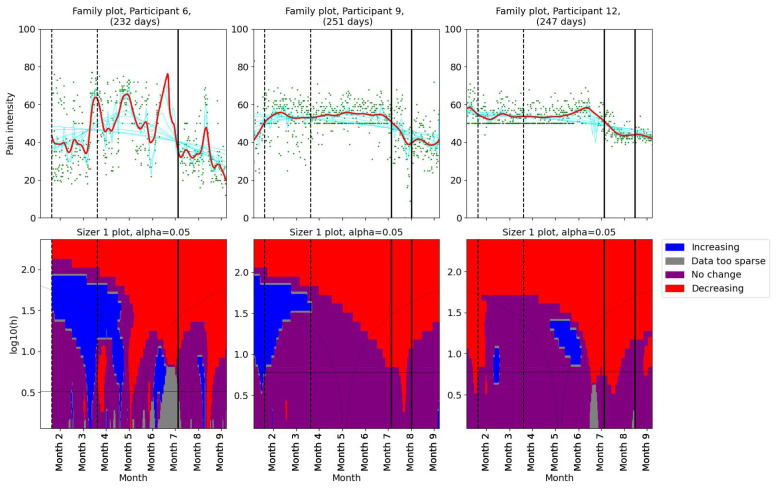
SiZer plots of the pain time series for participants 6, 9, and 12. The vertical dashed lines indicate the start and end of the baseline period, whereas the vertical solid lines are placed at the dates of the meetings with the physiotherapist. Explanation of the panels and colors is given in Section 2.4.

Pain levels were assessed using SiZer to identify significant changes. The results are presented in two groups. Three participants (1, 2, and 5) reported no decrease in pain intensity post-intervention (Figure 2). Participants 1 and 5 experienced a slight increase in pain after their first consultation with the physiotherapist. Participants 9 and 12 reported decreases in pain coinciding with the dates of their consultations with the physiotherapist (Figure 3). Further, they both experienced additional small decreases in pain following their follow-up appointments. Participant 12 also noted reduced headache and general pain during the second meeting. Participant 6 experienced a decrease in pain that began before her scheduled meeting with the physiotherapist, suggesting that other factors may have contributed to her pain relief. Participant 4, who had a single consultation, did not show a further decrease in pain post-meeting; however, her pain levels were already low prior to the meeting due to successful independent intervention (Figure 1). The results indicate a mixed response to physiotherapist consultations. While two participants (9 and 12) showed reductions in pain levels corresponding to their consultation dates, others (1, 2, and 5) did not benefit in terms of pain reduction.

#### 3.2.2. Changes in Activity Patterns

**Figure 4:**
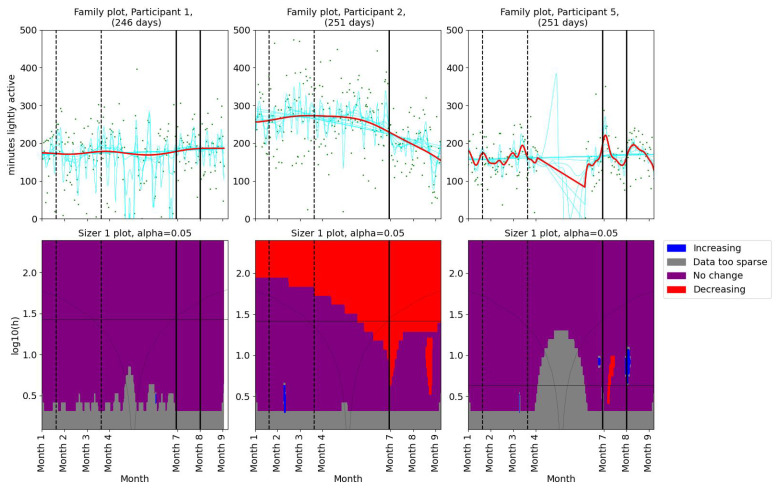
SiZer analyses of minutes of light activity per day for participants 1, 2, and 5. The vertical dashed lines indicate the start and end of the baseline period, whereas the vertical solid lines are placed at the dates of the meetings with the physiotherapist. Explanation of the panels and colors is given in Section 2.4.

**Figure 5:**
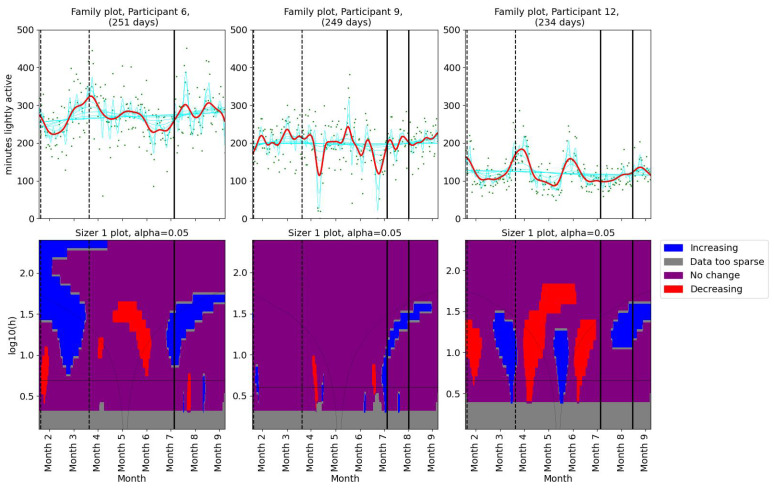
SiZer analyses of minutes of light activity per day for participants 6, 9, and 12. The vertical dashed lines indicate the start and end of the baseline period, whereas the vertical solid lines are placed at the dates of the meetings with the physiotherapist. Explanation of the panels and colors is given in Section 2.4.

As for the pain levels, we used SiZer analysis to assess whether the activity patterns changed post-intervention in the two groups of patients. Daily minutes of light activity for participants 1, 2, and 5 are shown in Figure 4. Participant 1 showed no significant change in her daily minutes of light activity throughout the study period. For Participant 2, there was a decrease in daily minutes of light activity before the physiotherapist consultation. A significant decrease in light activity minutes was observed in Participant 5 after the first meeting. Additionally, the family plot showed a decrease after the second meeting, although this change was not statistically significant. SiZer analyses of daily minutes of light activity for the second group (participants 6, 9, and 12) are shown in Figure 5. Two participants (6 and 9) showed a slight increase in light activity minutes per day before their first meeting with the physiotherapist. Participant 12 demonstrated an increase in light activity minutes between the two meetings, suggesting a positive adjustment in activity levels during the intervention. The SiZer analysis of daily minutes of light activity revealed varied responses among participants. While some participants increased their activity levels, others either decreased or showed no significant change.

In summary, the analysis of pain and activity levels throughout the study indicates a complicated relationship between these variables in FM patients. Participants who showed an increase in light activity levels, such as participants 6, 9, and 12, tended to achieve improvements or stability in pain levels. Conversely, participant 5, who demonstrated a significant decrease in time spent lightly active, also experienced a pain increase.

#### 3.2.3. Results From the Follow-Up Meeting

Four participants (1, 5, 9, and 12) attended a follow-up meeting with the physiotherapist, scheduled 4–6 weeks after the initial consultation. The follow-up aimed to assess the participants’ adherence to personalized activity plans, monitor progress, and address any challenges encountered:

- Participant 1 reported feeling unwell and bad sleep quality.
- Participant 5 reported that she had difficulties in her private life and did not prioritize physical activity.
- Participant 9 reported that she was not comfortable with walking faster. She agreed to continue being physically active and swimming.
- Participant 12 reported decreased headaches, decreased pain, and feeling better with more physical activity.

The feedback received from the follow-up meetings highlights different challenges and successes among participants. For example, Participant 12’s reporting of decreased headache and pain after increasing physical activity aligns with the hypothesis that physical activity can reduce pain in FM patients. Participant 5’s lack of compliance highlights the impact of personal circumstances, indicating the need for flexible and adaptive interventions.

### 3.3. Answers From Questionnaire

**Figure 6:**
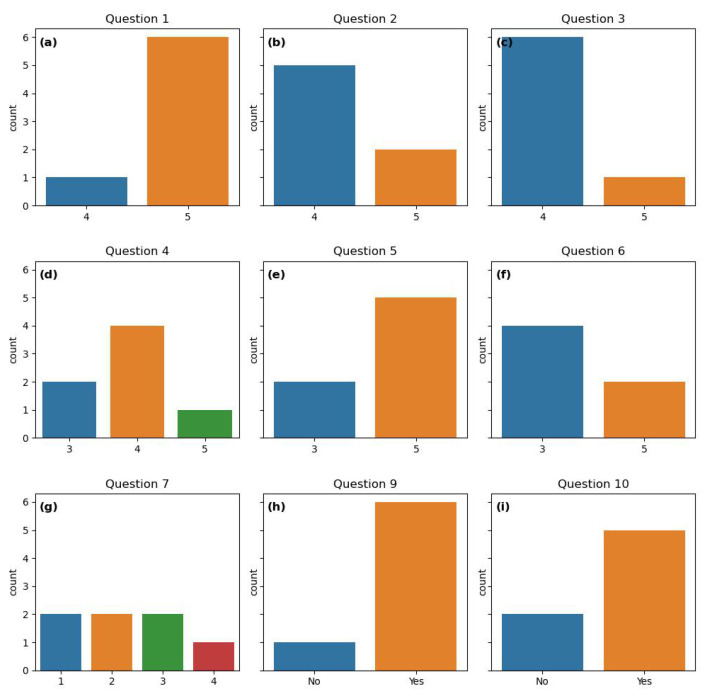
Count plots of the questionnaire response to questions answered by “yes”/“no” or using the 5-point Likert scale. All the questions are listed in Section 2.5. (**a**) Response to question 1. (**b**) Response to question 2. (**c**) Response to question 3. (**d**) Response to question 4.(**e**) Response to question 5. (**f**) Response to question 6. (**g**) Response to question 7. (**h**) Response to question 9. (**i**) Response to question 10.

Visualizations of the questionnaire response can be found in Figure 6. In Appendix A, the entire questionnaire together with the number of participants choosing the different alternatives is presented. For question 1, six participants chose the highest score, “Comfortable and would like to continue” (Figure 6a), indicating a high comfort level among participants regarding wearing a Fitbit tracker and registering pain. One participant was comfortable, but would not like to continue after the end of the project. Participants reported overall satisfaction with the Fitbit tracker and the pain registration app (questions 2 and 3) (Figure 6b,c). Here, all participants responded “satisfied” or “very satisfied”.

All participants were comfortable with digital physiotherapist consultations, although four of them would prefer physical meetings (question 4). Two were neutral, i.e., comfortable with both, and one preferred digital meetings (Figure 6d).

Five participants felt comfortable following the personalized activity plan and would like to continue. Two were neutral (Figure 6e). Regarding the visualizations of pain and activity patterns, two participants found them very useful, four were neutral and one did not receive graphs due to lack of Fitbit data for this participant (Figure 6f).

The perception of the effectiveness of the varied widely (Figure 6g). One participant agreed with the following affirmation: “I think that the personalized activity plan that I followed has helped me improve my pain management”. Four participants strongly disagreed or disagreed, whereas two were neutral.

Questions 9 and 10, about the desire to continue the intervention and producing data, are answered by “yes” or “no”. Six and five participants responded “yes” to these questions, respectively (Figure 6h,i). This indicates that most participants are interested in continuing both the intervention and in producing data. Six participants would like to continue the intervention. Five participants were willing to continue producing data, although the physiotherapist would not be available anymore.

Participants expressed a strong appreciation for being involved in the research and recognized the need for more research on chronic pain management. Feedback included requests for exploring other pain management methods and enhancing follow-up support.

The quantitative analysis of the questionnaire response highlights the strengths of our study, such as high satisfaction with the Fitbit tracker, pain registration app, and personalized activity plans. Although most of the participants were comfortable with the physical activity plans and would like to continue following them, only 1 participant agreed that the plan was helpful for her pain management. The variability in some responses underscores the need for personalized pain management tools.

## 4. Discussion

In addition to the patients who started regular physical activity independently, three out of six participants experienced a reduction in pain during the intervention. According to Fitbit data, these three participants also increased their time spent being “lightly active”. This finding is consistent with previous studies that conclude that physical activity is beneficial for FM patients. For the other three participants, the intervention was not successful. We investigated the possible causes and we noticed that for different reasons those participants were not able to properly follow the activity recommendations. For example, one participant contracted COVID-19 during the intervention and was unable to be more active. This is confirmed by the questionnaire results where two participants reported that they were unable to follow their physical activity plan. Participants 1 and 5 reported having difficulties in their daily life during the meetings with the physiotherapist. Our intervention lasted less than two months. The patients may need more time to implement lifestyle changes and experience the effect of the suggested physical activity program. This was reported explicitly in the feedback we received from the participants. Longer interventions would also allow adjusting the goals in terms of physical activity over time, based on the obtained results. A previous study performed an intervention on patients with chronic widespread pain supervised by a physiotherapist [55]. Moreover, 139 individuals were randomized to an intervention group and a control group. The intervention lasted 6 months and the support was provided digitally. According to the results, there were no significant differences in pain intensity between the groups after 6 months. The discussion of the paper states that a mean increase in physical activity of 2 hours per week might not be enough to have an impact on pain intensity.

It is clear from the questionnaire answers that the participants found the project meaningful, even though not all of them experienced improvement in pain. One participant commented that what mattered the most was that finally they had been heard and taken seriously. Receiving attention may be beneficial for pain management. We observed an unexpected result during the period when the participants were waiting for the intervention: participants reported experiencing pain relief even though the intervention had not started yet. This is surprising given that previous research has suggested that waiting for treatment can actually increase symptoms [56]. One possible explanation for this unexpected finding is that when our participants were informed that they would start an intervention, they may have developed positive expectations that they would feel better. These positive expectations could potentially have produced a placebo effect that contributed to the observed pain relief. Further research is needed to investigate this hypothesis.

In this study, the relationship between the number of consultations with the physiotherapist and pain reduction did not show a clear pattern. To be specific, among participants who had two consultations, pain reduction was observed in only half (Participants 9 and 12), suggesting that the number of meetings alone may not be crucial for pain relief. This finding suggests that other influencing factors are more important for the outcome, including individual adherence to the activity recommendations, personal motivation, and baseline health status.

Four participants showed a significant positive correlation between resting heart rate and pain intensity. It is well known that a low (high) resting heart rate is related to good (worse) physical shape. This finding suggests that there might be an indirect relationship between resting heart rate and pain. A hypothesis worth further investigation is that a lower resting heart rate is associated with less pain in people with FM. Two participants showed a significant negative correlation between time in certain activity zones and pain. Also, one participant had a significant positive correlation between sedentary time and pain. These findings are consistent and support the hypothesis that physical activity is beneficial for FM patients. By collecting data from a larger sample size and over an even longer period of time, potentially stronger correlations may be found.

An important strength of this work is that we have data gathered over a long period of time. Longitudinal data allow us to observe the effects of lifestyle changes or other interventions. Long-term data also help in understanding temporal patterns and trends in activity and pain. Furthermore, the collected pain data have a high resolution (three measures per day). High-resolution data can reveal insights that might be missed with coarser data. The data can help in understanding the micro-patterns and triggers of pain. With detailed pain profiles, treatments can be better tailored to the individual’s specific needs and responses. For physical activity, the collection of high-resolution data is made possible by activity trackers. The use of activity trackers instead of relying solely on self-reported physical activity presents numerous opportunities. These devices can potentially provide more information on the subject’s activity due to their ability to monitor continuously and non-intrusively. Additionally, the cost of activity trackers is not prohibitively expensive, making them a viable option for extensive use in studies or personal health monitoring over long periods.

Improving chronic pain management would also represent a benefit for the entire society. Diseases affecting muscles, skeleton, and connective tissue represent the second largest cost to the healthcare system, following mental diseases, according to a report from the Norwegian Directory of Health [57]. In 2015, the total cost related to muscle, skeleton, and connective tissue diseases was USD 22.9 billion. In 2024, this corresponds to more than USD 30 billion.

Our results suggest that it may be possible to reduce pain for FM patients using regular and personalized physical activity, which might reduce the societal cost of FM. According to Praksisnett (an infrastructure for research in primary care [58]), in Norway, approximately 100,000 FM patients visit their general practitioner every year, corresponding to 1.84% of the Norwegian population. We believe that regular physical activity could help a percentage of those patients improve their health and quality of life, decreasing the need for visiting the general practitioner’s office. As a result, this could reduce the cost for our society, while saving time for healthcare personnel.

In the future, we will aim to extend this work by several means. First of all, we will aim to include more variables than pain in the registration app, such as stress level and other psychological variables. All FM patients in our group agreed that stress can trigger pain for them. This statement is supported by a systematic review [59]. A potential problem with including more variables is that we might end up asking for too much information. If the registration process burdens the participants, they may drop out. Possible solutions include making those variables optional to register and implementing strategies to minimize dropouts. A systematic review [60] reviewed randomized control trials in order to find dropout rates. One conclusion was that interventions should be supervised by an expert (for example a physiotherapist) to minimize the risk of dropout.

We will also aim to extend the duration of the intervention, to provide more insights on the sustainability over time of the recommended activities. An extended intervention can also help track long-term benefits and provide ongoing support to the patients. In addition, a longer follow-up may allow us to find the intensity that works best for the individual FM patient. This is supported by the questionnaire results, where one participant stated that there was not enough follow-up. To improve follow-up, we will also consider increasing the duration of the meetings with the physiotherapist and scheduling regular follow-up appointments. We will also consider facilitating support groups where patients can share their experiences, challenges, and successes.

While standardized interventions make it easy to compare results across a larger cohort, they may not fully address individual differences in patient needs and preferences. Conversely, personalized interventions are tailored to meet the specific conditions and preferences of each patient. In our study, the choice to implement an individualized approach was driven by the nature of FM, which affects patients differently. Furthermore, the diversity of our patient group and the findings from the literature [41,42,43,45] suggested that a personalized approach could be more promising. The limited sample size also made a personalized approach more natural.

## 5. Limitations

Implementing a study like the one described in this manuscript presents several questions. Using patient-generated data will always include challenges. In our case, the user might forget to register their pain levels, although the app can provide reminders. Missing data is also a problem in data collected from activity trackers. When the user does not wear the tracker, for example, while it is charging, it results in missing data. The user must also remember to synchronize the Fitbit to the Fitbit cloud. Otherwise, data will be lost as the Fitbit device is not able to store large amounts of data. Additionally, the accuracy of activity trackers, as noted in studies like [61,62], means that data quality is always a concern. Challenges related to the use of Fitbit in physical activity interventions are presented in [63], together with possible solutions. Examples of challenges are selecting a suitable tracker model, setting up the data collection environment, and participants’ unfamiliarity with Fitbit technology. In addition to missing data, bias can affect data collection. For example, one participant said that registering pain made her more aware of her pain. She looked forward to not having to register her pain three times per day. This might lead to bias in the pain data. Recruiting large groups of patients also poses a significant challenge, as does the need for clinical experts to supervise the participants. Moreover, having a dedicated contact person to manage interactions with the participants is essential. Keeping participants engaged over the long term is crucial; in this study, engagement was maintained through regular meetings and by presenting participants with visualizations of their data.

An obvious objection to the work done in this paper is that our sample size is small. However, for each individual, the follow-up was long enough to gain insight into their pain and activity profile over time. We acknowledge that the small sample size makes us unable to generalize our findings and perform comparative statistical tests across participants. However, our work could be a stepping stone to a randomized controlled trial with a sufficiently large sample size. The intervention group should be provided with personalized activity recommendations, whereas the control group should receive a homogeneous movement program. In this way, one can assess whether a personalized program reduces pain more efficiently than a standard activity program. A pilot study [64] provided nine women with FM individual health and wellness coaching. The coaching protocol consisted of biweekly private telephone sessions and 18 group telephone sessions over a period of 12 months. This study found significant pain reduction in the participants and concluded that health and wellness coaching has the potential to improve FM patients’ health. The authors also state that they want to conduct a controlled trial with a larger sample size to expand on the work done in the pilot study. Another pilot study [65] tested resistance exercise as an intervention for ten women with FM. The result from [65] was that the participants preferred heavy load resistance exercise over light or moderate resistance exercise. The authors suggest that a similar intervention should be tested on a larger group of patients.

This study involved only female participants, which was not our intention, but rather a result of the recruitment process. All respondents to our study invitation were women, which limited the gender diversity. This limitation is relevant given the potential differences in how men and women may experience and manage FM symptoms. Future research should aim to engage male patients more efficiently, possibly by exploring different recruitment strategies.

## 6. Conclusions

Below is a summary of our findings regarding the research questions raised in Section 2.4:

1. Does spontaneous physical activity affect the pain pattern in our research participants?
  - For four participants, we detected a significant positive correlation between pain and resting heart rate. This might indicate that their physical condition affects their pain levels.
  - One of the patients in this study started regular activity spontaneously, resulting in a significant improvement in pain management.
2. Can the results of one patient’s successful pain reduction intervention be replicated for other FM patients?
  - Three participants showed a reduction in pain while they increased their time spent lightly active per day. This suggests that personalized activity recommendations can be helpful for FM patients.
  - Three participants did not experience a reduction in pain during the intervention. However, for personal reasons, they were not able to follow the recommendations.

Although the intervention was not successful for all participants in terms of pain relief, they expressed gratitude for being part of this study. Our findings suggest that a personalized approach to physical activity could be part of treatment plans for FM patients. However, further research involving a larger cohort over a longer period is needed to validate these preliminary findings.

It is important to consider our findings in the context of the limitations, including the small sample size and the short duration of the intervention. These factors may limit the possibility of generalizing the results.

Our research has elucidated the potential for using wearable technology to monitor physical activity in FM patients. Future studies could assess other variables measured by wearables (such as sleep patterns) in relation to FM symptoms, to investigate further correlation between lifestyle and pain profile.

We believe that the work described in this manuscript can be extended to a larger patient group and potentially improve the health and quality of life of several FM patients.

## Figures and Tables

**Table 1:** Significant results from Spearman’s correlation test on different Fitbit variables and maximum pain during one day.

Participant	Variable	Test Statistic	*p*-Value	# Values
Participant 1	Resting heart rate	0.256	<0.001	333
Participant 2	Minutes lightly active	−0.265	<0.001	254
Participant 2	Steps	−0.247	<0.001	254
Participant 2	Resting heart rate	0.334	<0.001	254
Participant 9	Minutes spent sedentary	0.234	<0.001	246
Participant 9	Resting heart rate	0.167	0.005	238
Participant 12	Minutes spent very active	−0.120	0.036	224
Participant 12	Resting heart rate	0.419	<0.001	223

## Data Availability

The data are not publicly available due to privacy issues (patient data). We do not have consent from the participants to share the data.

## Abbreviations

The following abbreviations are used in this manuscript:

FM	fibromyalgia
VAS	visual analog scale
SiZer	significant zero crossings of derivatives

## Appendix A

### Questionnaire

In this appendix, we include the full questionnaire with all possible choices for each question, as well as the number of participants who chose different alternatives (in parentheses).

1. How did you feel about wearing a Fitbit and registering pain intensity 3 times a day for the duration of this project?
  1 It felt very uncomfortable, and I stopped doing it during the project.
  2 It felt uncomfortable, but I managed to do it for the duration of the project.
  3 Neutral.
  4 I was comfortable doing it for the duration of the project, but I do not feel like continuing to register/collect data for a longer time (1 participant).
  5 I felt comfortable doing it for the duration of the project, and I would not mind continuing to collect data. (6 participants)
    I do not want to answer.
2. How would you rate your level of satisfaction with the Fitbit tracker?
  1 Very dissatisfied.
  2 Dissatisfied
  3 Neutral: neither satisfied nor dissatisfied
  4 Satisfied (5 participants)
  5 Very satisfied (2 participants)
    I do not want to answer.
3. How would you rate your level of satisfaction with the pain registration app?
  1 Very dissatisfied.
  2 Dissatisfied
  3 Neutral: neither satisfied nor dissatisfied
  4 Satisfied (6 participants)
  5 Very satisfied (1 participant)
    I do not want to answer.
4. How did you feel about the fact that the meeting with the physiotherapist was digital, instead of in person?
  1 It felt uncomfortable and I would not do it again.
  2 It felt uncomfortable, but I would do it again if it was the only option.
  3 Neutral: no preferences when it comes to digital VS in-person; I am comfortable with both (2 participants).
  4 I was comfortable with the digital meeting, but I still prefer in-person meetings (4 participants).
  5 I felt very comfortable with it being digital: I prefer digital meetings over in-person meetings (1 participant).
    I do not want to answer.
5. How did you feel about the physical activity plan that you have created with the physiotherapist?
  1 It felt very uncomfortable and I stopped following the activity plan before the end of the project.
  2 It felt uncomfortable, but I managed to follow the activity plan, just to complete the project.
  3 Neutral (2 participants).
  4 I felt comfortable following my activity plan for the duration of the intervention, but I do not feel like continuing.
  5 I felt comfortable following my activity plan for the duration of the intervention, and I would not mind continuing (5 participants).
    I do not want to answer.
6. Was it useful to discuss the graphs that were provided during the meeting?
  1 Totally useless.
  2 Useless.
  3 Neutral (4 participants).
  4 Useful.
  5 Very useful (2 participants).
    Did not receive graphs due to lack of Fitbit data (1 participant).
7. Do you agree with this affirmation: “I think that the personalized activity plan that I followed has helped me improve my pain management”?
  1 Strongly disagree (2 participants).
  2 Disagree (2 participants).
  3 Neutral (2 participants).
  4 Agree (1 participant).
  5 Strongly agree.
    I do not want to answer.
  [If the answer to question 4 is 1 or 2]
8. What do you think are the reasons the personalized activity plan did not work for you? You can select several answers
  - I was not able to follow it due to reasons that are **not** related to pain or FM (e.g., I did not have time for it, I was sick) (2 participants).
  - I was not able to follow it due to reasons related to pain or FM.
  - The duration was too short: I think it may work if I continue following it (1 participant).
  - I was not satisfied with my personalized activity plan, but I think that a different activity plan could work
  - I do not think that exercising regularly can help with pain management (1 participant).
  - Other (free text)
    – I had a good plan in place already.
  Would you like to elaborate on your answer? (Free text)
  - I do not think that physical activity can help with pain management. But if my general health can be improved by other methods, physical activity might contribute to maintaining my health.
  - Light physical activity has been helpful for my pain management.
  - I have several strategies for pain management; physical activity, stress reduction, good sleep quality, and the right diet.
9. If offered, would you like to continue with the “assisted” personalized activity intervention?
  - Yes (6 participants).
  - No (1 participant).
  Would you like to elaborate on your answer? (Free text)
  - I found it valuable to contribute to better health and quality of life for future chronic pain patients.
  - I think this is an interesting project. More research on chronic pain is needed.
  - I think it is important to contribute to research on chronic pain.
10. We are considering keeping the app up and running for 6 extra months. It would be possible to register pain 3 times a day and collect Fitbit data. However, consultations with the physiotherapist would not be available. Would you be willing to collect data for us?
  - Yes (5 participants).
  - No (2 participants).
11. Help us improve the project! Do you have any input to share with us?
